# Association of autoimmune and allergic diseases with senile cataract: a bidirectional two-sample Mendelian randomization study

**DOI:** 10.3389/fimmu.2024.1325868

**Published:** 2024-03-22

**Authors:** Weichen Yuan, Xiangrui Li, Guan Wang, Bo Qu, Fangkun Zhao

**Affiliations:** ^1^Department of Ophthalmology, The Fourth Affiliated Hospital of China Medical University, Shenyang, China; ^2^Key Lens Research Laboratory of Liaoning Province, Shenyang, China

**Keywords:** autoimmune diseases, allergy, Mendelian randomization, senile cataract, GWAS - genome-wide association study

## Abstract

**Background:**

Many observational studies have been reported that patients with autoimmune or allergic diseases seem to have a higher risk of developing senile cataract, but the views are not consistent. In order to minimize the influence of reverse causality and potential confounding factors, we performed Mendelian Randomization (MR) analysis to investigate the genetic causal associations between autoimmune, allergic diseases and senile cataract.

**Methods:**

Single nucleotide polymorphisms associated with ten common autoimmune and allergic diseases were obtained from the IEU Open genome-wide association studies (GWAS) database. Summary-level GWAS statistics for clinically diagnosed senile cataract were obtained from the FinnGen research project GWAS, which consisted of 59,522 individuals with senile cataracts and 312,864 control individuals. MR analysis was conducted using mainly inverse variance weighted (IVW) method and further sensitivity analysis was performed to test robustness.

**Results:**

As for ten diseases, IVW results confirmed that type 1 diabetes (OR = 1.06; 95% CI = 1.05-1.08; *p* = 2.24×10^-12^), rheumatoid arthritis (OR = 1.05; 95% CI = 1.02-1.08; *p* = 1.83×10^-4^), hypothyroidism (OR = 2.4; 95% CI = 1.42-4.06; *p* = 1.12×10^-3^), systemic lupus erythematosus (OR = 1.02; 95% CI = 1.01-1.03; *p* = 2.27×10^-3^), asthma (OR = 1.02; 95% CI = 1.01-1.03; *p* = 1.2×10^-3^) and allergic rhinitis (OR = 1.07; 95% CI = 1.02-1.11; *p* = 2.15×10^-3^) were correlated with the risk of senile cataract. Celiac disease (OR = 1.04; 95% CI = 1.01-1.08; *P* = 0.0437) and atopic dermatitis (OR = 1.05; 95% CI = 1.01-1.10; *P* = 0.0426) exhibited a suggestive connection with senile cataract after Bonferroni correction. These associations are consistent across weighted median and MR Egger methods, with similar causal estimates in direction and magnitude. Sensitivity analysis further proved that these associations were reliable.

**Conclusions:**

The results of the MR analysis showed that there were causal relationships between type 1 diabetes, rheumatoid arthritis, hypothyroidism, systemic lupus erythematosus, asthma, allergic rhinitis and senile cataract. To clarify the possible role of autoimmune and allergy in the pathophysiology of senile cataract, further studies are needed.

## Introduction

Senile cataract, also known as age-related cataract, is one of the leading causes of treatable blindness in the world, affecting 17% of the global population (1). Senile cataract is the most common type of cataract among adults, with onset between ages of 45 and 50. Even with the rapid development of cataract surgery, senile cataract still causes a huge disease and economic burden, especially in developing countries (2). Identifying potential risk factors to determine the mechanism of cataract formation and preventive methods is therefore of paramount importance. Currently, risk factors such as ageing, smoking, alcohol consumption, hypertension, and diabetes have been proved to contribute to the occurrence of cataract (3–7). However, the influence of immune related diseases as risk factors on cataract is not well studied.

Protecting the host from infection is the primary function of the immune system. The inability to distinguish self from non-self is often referred to as a breach of tolerance and is the underlying mechanism for autoimmune disease. The overall prevalence of autoimmune diseases in the general population is in the range of 3-5% (8). There are almost 100 different types of autoimmune diseases, the most common of which are autoimmune thyroid disease and type 1 diabetes (T1D) (9). The incidence of allergic diseases, represented by asthma, atopic dermatitis (AD) and allergic rhinitis (AR) , has increased dramatically in the past three decades, and now affects approximately 20% of the population, becoming a public health problem that imposes a heavy burden on society (10). Evidence from previous observational studies suggests that some autoimmune and allergic diseases, such as celiac disease (CeD) (11), systemic lupus erythematosus (SLE) (12), T1D (13), multiple sclerosis (MS) (14), psoriasis (15), asthma (16) and AR (17) may increase the risk of cataracts. These observational studies tend to be susceptible to selection bias, residual confounders and reverse causation. Thus, assessing the causal relationship between autoimmune and allergic diseases and the development of senile cataract can provide clues for the etiology of senile cataract.

Mendelian randomization (MR), as an epidemiological approach, has been widely used to evaluate the potential causal association between exposures and disease results (18). This approach minimizes residual confounding because genetic variants are randomly assembled at the time of conception and are therefore independent of personal lifestyle and environmental factors (19). At the same time, the interference of reverse causality can also be avoided (20). Compared with the gold standard randomized controlled trial (RCT) that established causality, MR used data from large-scale GWAS which is timelier and the sample size is larger. In addition, sometimes randomized controlled trials cannot be conducted because they are costly, unfair, and even unethical. MR studies can overcome these shortcomings while results are broadly consistent with RCTs (21).

Lens epithelial cells are the most active metabolic cells in the lens, which undergo oxidation, insolubility and cross-linking during cataract formation. These cells then migrate to the lens equator to form lens fibers, which are gradually compressed in the center, resulting in hardening and opacity of the lens nucleus (22). The pathophysiological mechanism of lens opacity in cataract is usually attributed to oxidative stress (23). Studies on the mechanisms of autoimmunity or allergy in patients with cataract are not common and have only been reported in a few publications (24–26). It is still unclear whether autoimmune, allergic diseases and senile cataract are linked through a shared genetic etiology. To our knowledge, there are currently no MR study evaluating the association between autoimmunity, allergic disease and senile cataract. Through this study, it is possible for us to reveal the genetic characteristics and immune related biological processes associated with senile cataract, bridging the significant knowledge gap about the complex causes of this disease.

## Methods

### MR assumptions and study design

**Figure 1:**
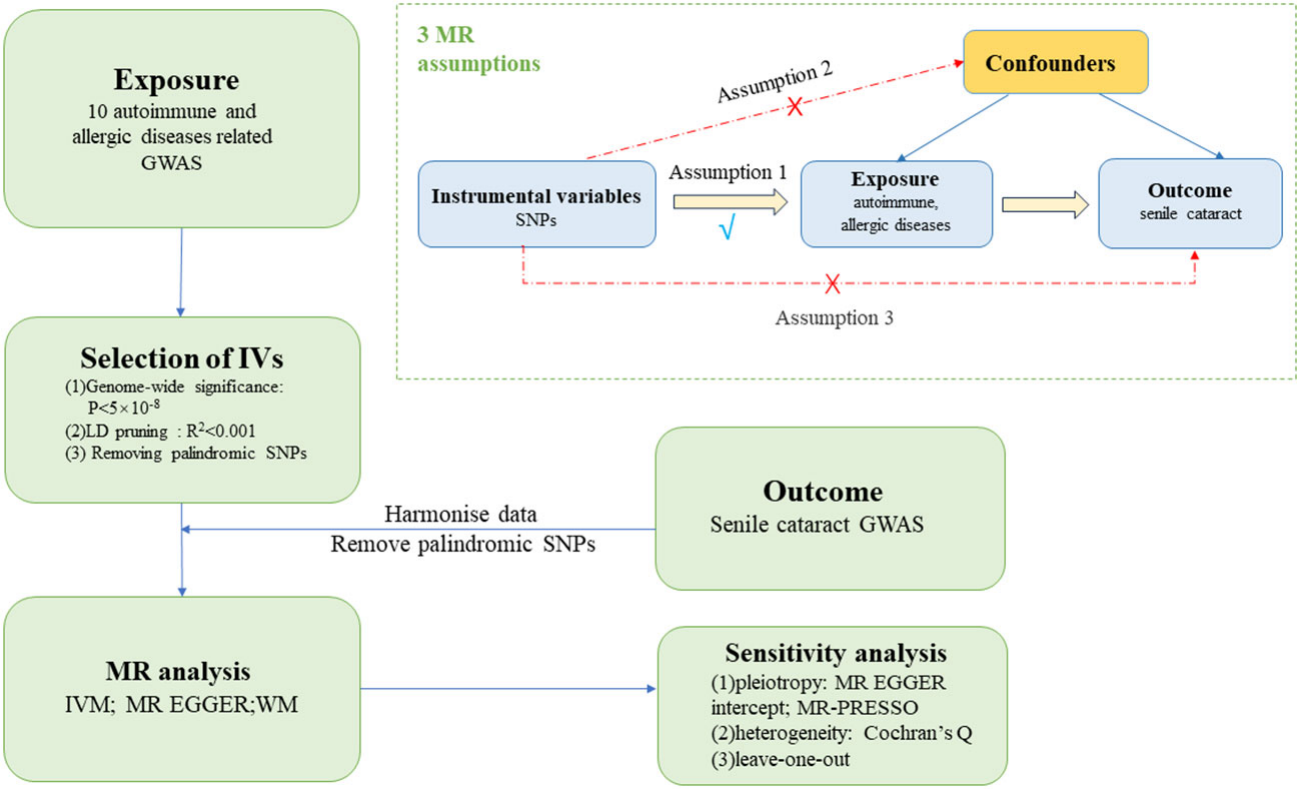
The flow chart of MR research and three MR assumptions.

T1D, rheumatoid arthritis (RA), hypothyroidism, SLE, CeD, MS, psoriasis, asthma, AR and atopic dermatitis (AD) included in our study were determined according to previously published observational studies. In order to assess the causal connections between senile cataract and these diseases, we conducted a two-sample MR analysis. Summary-level data from the GWASs were obtained for autoimmune, allergic diseases and senile cataract. In order to obtain reliable results, the MR analysis meets the following three assumptions (1) instrumental variables (IVs) finally included in the use must be closely related to autoimmune or allergic diseases; (2) IVs and confounding factors (affecting autoimmune, allergic diseases and senile cataract) were independent of each other; (3) IVs only affect senile cataract only through autoimmune or allergic disease. **Figure 1** shows the flow chart of MR research between autoimmune, allergic diseases and senile cataract and three MR assumptions. To minimize bias due to ethnic stratification, we restricted included individuals to European population.

### Exposure sources of autoimmune and allergic diseases

Summary level data for all 10 exposures were obtained from Integrative Epidemiology Unit (IEU) Open GWAS database (https://gwas.mrcieu.ac.uk/). We prioritized using the GWAS dataset with the largest samples size as exposures. If any of the following three situations occur, we will select other relatively large GWAS data: 1) insufficient instrumental variables; 2) racial differences or sample overlap; 3) significant pleiotropy in preliminary analysis. Ultimately, summary GWAS data for hypothyroidism and psoriasis were from UK Biobank. The GWAS summary data for T1D (27), RA (28), CeD (29), SLE (30), MS (31), asthma (32), AR (33) and AD (34) were abstracted from different publicly available GWASs. Detailed information for the data sources was presented in **Table 1**.

**Table 1:** Sources and characteristics of exposure.

Exposure	GWAS-ID	Author, Journal/Consortium	Sample size	PMID
Type 1 diabetes	ebi-a-GCST90018925	Sakaue et al., Nat. Genet.	457,695	34594039
Rheumatoid arthritis	ebi-a-GCST005569	Eyre et al., Nat. Genet.	47,580	23143596
Hypothyroidism	ukb-a-77	UK Biobank	337,159	NA
Systemic lupus erythematosus	ebi-a-GCST003156	Bentham et al., Nat. Genet.	14,267	26502338
Celiac disease	ieu-a-1060	Dubios et al., Nat. Genet.	11,950	20190752
Multiple sclerosis	ebi-a-GCST001198	Sawcer et al., Nature	26,621	21833088
Psoriasis	ukb-a-100	UK Biobank	337,159	NA
Asthma	ebi-a-GCST90014325	Valette et al., Commun Biol.	408,442	34103634
Allergic rhinitis	ebi-a-GCST90013920	Mbatchou et al., Nat. Genet.	407,746	34017140
Atopic dermatitis	ebi-a-GCST90027161	Sliz et al., J Allery Clin Immunol.	796,661	34454985

A quality check of the single nucleotide polymorphisms (SNPs) is performed to meet the basic assumptions of MR: (1) SNPs associated with autoimmune diseases reached the genome-wide significance threshold (P<5×10^-8^). (2) We further clumped the SNPs in linkage disequilibrium (LD) analysis (R^2^ < 0.001, clumping distance = 10,000kb). (3) The palindromic SNPs with intermediate allele frequencies were eliminated. (4) When the original SNP was not available, proxy SNPs with r^2^ > 0.9 according to LD link (https://ldlink.nci.nih.gov/) were used. To ensure robust associations between instrumental and endogenous variables and to prevent weak instrumental variable bias, we calculated *R*^2^ [*R*^2^ = 2 × EAF × (1 − EAF) × *b*^2^], representing the proportion of variation explained by instrumental variable SNPs. Simultaneously, we performed calculations of the F-statistic [*F* = *R*^2^ × (*N* − 2) / (1 − *R*^2^)] to assess the potency of IVs, whereby IVs with an F-statistic exceeding 10 are deemed to be valid (35).

### Outcome sources of senile cataract

To reduce bias due to sample overlap, the summary statistics for the senile cataract GWAS were selected from the FinnGen research project (https://r9.finngen.fi/), including 59,522 cases of senile cataracts and 312,864 cases of population controls. This study defines senile cataract by H25 of the International Classification of Disease-10 (36).

All analyses were based on publicly shared databases and no additional ethical approvals were required.

### Statistical analysis

#### MR analysis

The “TwoSampleMR” R package (version 0.5.7) was used for bidirectional univariable two-sample MR analysis between autoimmune, allergic diseases and senile cataract. Inverse variance weighted (IVW) methods were applied to evaluate the causality between autoimmune, allergic disease-related IVs and senile cataract risk, as the IVW approach is most effective in terms of statistical power when all IVs were valid and there is no horizontal pleiotropy (37). Cochrane's Q test was applied to test whether heterogeneity existed, and if so, an IVW random-effects model was used, otherwise an IVW fixed-effects model was used. The effect size is indicated by the odds ratio (OR) along with its 95% confidence interval (CI). However, even if only one genetic variation is invalid, the IVW method may provide biased estimates. In order to solve the robustness, two other methods are carried out, including weighted median (WM) method and MR-Egger test. WM gives a reliable estimate assuming that no less than 50% of the IVs are valid (35). The results of MR Egger remain valid when SNPs with pleiotropy were more than 50% (38).

#### Sensitivity analysis

If genetic variants have horizontal pleiotropy, our IVW results will be invalidated. Intercept terms obtained from the MR-Egger regression were used to evaluate imbalanced pleiotropic effects (39). The MR-Egger estimate would be equal to the IVW estimate if the intercept term was zero and the test *p*-value is greater than 0.05. The MR pleiotropy residual sum and outlier (MR-PRESSO) method (R package “MR-PRESSO” v1.0) can also detect the outliers that may possess the characteristic of horizontal pleiotropy and provide corrected estimates after removing outliers (40). Cochrane's Q test was used to test heterogeneity (41). If heterogeneity exists, we will use IVW random-effects model to calculate the main results. Leave-one-out analysis is conducted to assess the sensitivity of results to individual variants by sequentially excluding one SNP at a time to estimate whether results are biased or driven by individual SNPs (42). In order to reduce false positive rate in batch analysis, we use Bonferroni method for multiple testing. The threshold for statistical significance was defined as a *p*-value <5×10^-3^ (*p* =0.05 / (ten exposures × one outcome) adjusted for ten exposures and one outcome using the Bonferroni method. A p-value ranging from 0.005 to 0.05 was deemed to indicate suggestive significance.

## Results

### Selection of IVs associated with autoimmune diseases

In this study, we reported our MR analysis according to STROBE-MR (Strengthening the Reporting of Mendelian Randomization Studies) guidelines to improve the clarity, transparency and reproducibility of the study (**Supplementary Table S1**).

The number of SNPs ranged from 7 to 117, after quality control steps by LD effects and palindromic. The F-statistic of SNPs ranges from 29.4 to 2815.2, indicating that each SNP revealed adequate validity. The detailed information for each SNP and its R^2^ and F-statistic value were shown in **Supplementary Table S2**.

### MR analysis

**Figure 2:**
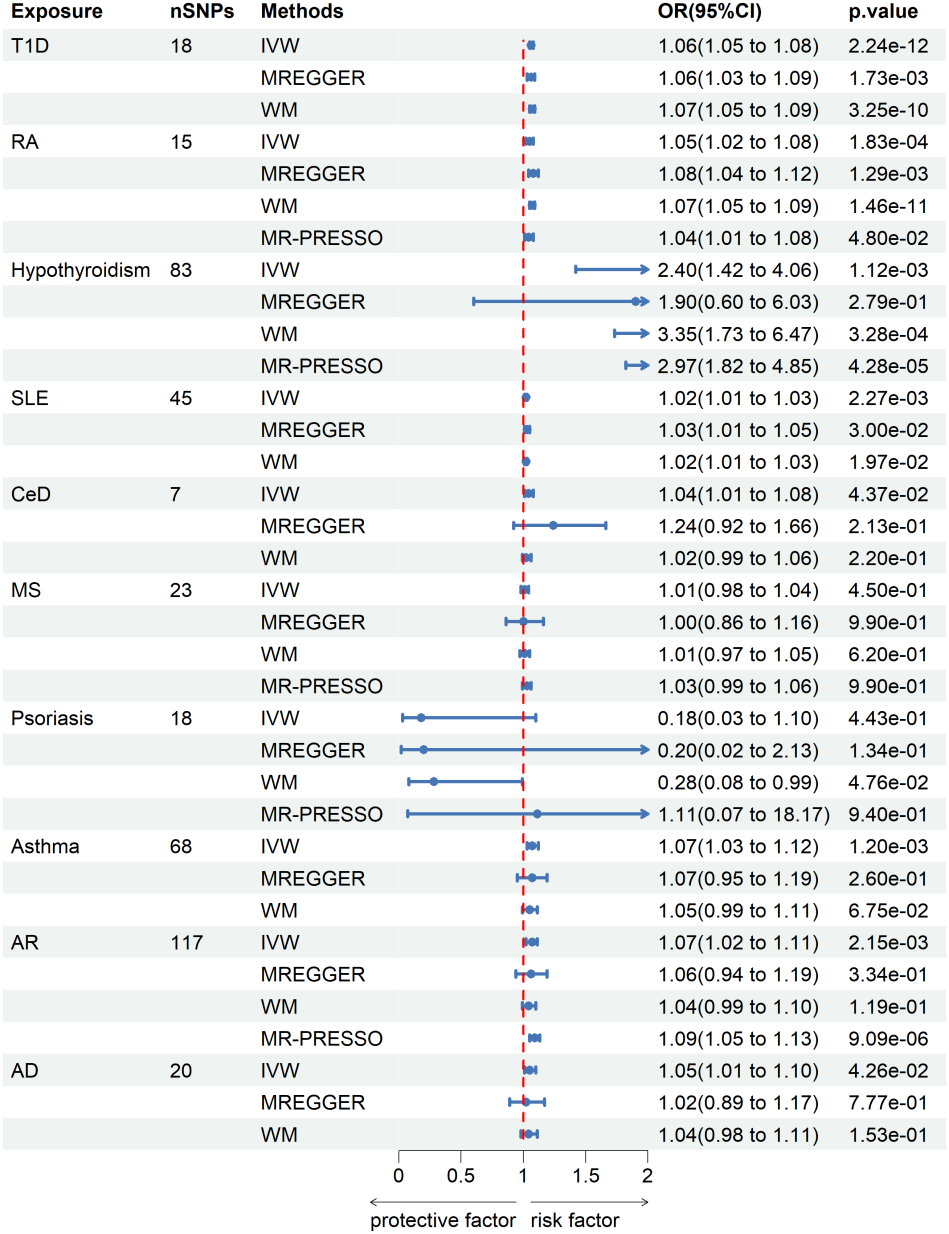
Mendelian randomization estimates from instrument variants for autoimmune and allergic diseases on risk of senile cataract.

IVW analysis showed that T1D (OR = 1.06; 95% CI = 1.05-1.08; *p* = 2.24×10^-12^), RA (OR = 1.05; 95% CI = 1.02-1.08; *p* = 1.83×10^-4^), hypothyroidism (OR = 2.4; 95% CI = 1.42-4.06; *p* = 1.12×10^-3^), SLE (OR = 1.02; 95% CI = 1.01-1.03; *p* = 2.27×10^-3^), asthma (OR=1.07; 95% CI = 1.03-1.12; *p* = 1.2×10^-3^) and AR (OR = 1.07; 95% CI = 1.02-1.11; *p* = 2.15×10^-3^) were causally associated with a significantly increased risk of senile cataract in European populations. CeD (OR = 1.04; 95% CI = 1.01-1.08; *P* = 0.0437) and AD (OR = 1.05; 95% CI = 1.01-1.10; *P* = 0.0426) exhibits a suggestive connection with senile cataract after Bonferroni correction. There was insufficient evidence to suggest that genetically predicted MS (OR = 1.01, 95% CI = 0.98-1.04; *P* = 0.45) and psoriasis (OR = 0.18; 95% CI =0.03-1.1; *P* =0.443) were associated with senile cataracts. These associations are consistent across WM and MR Egger methods, with similar causal estimates in direction and magnitude (**Figure 2**).

**Figure 3:**
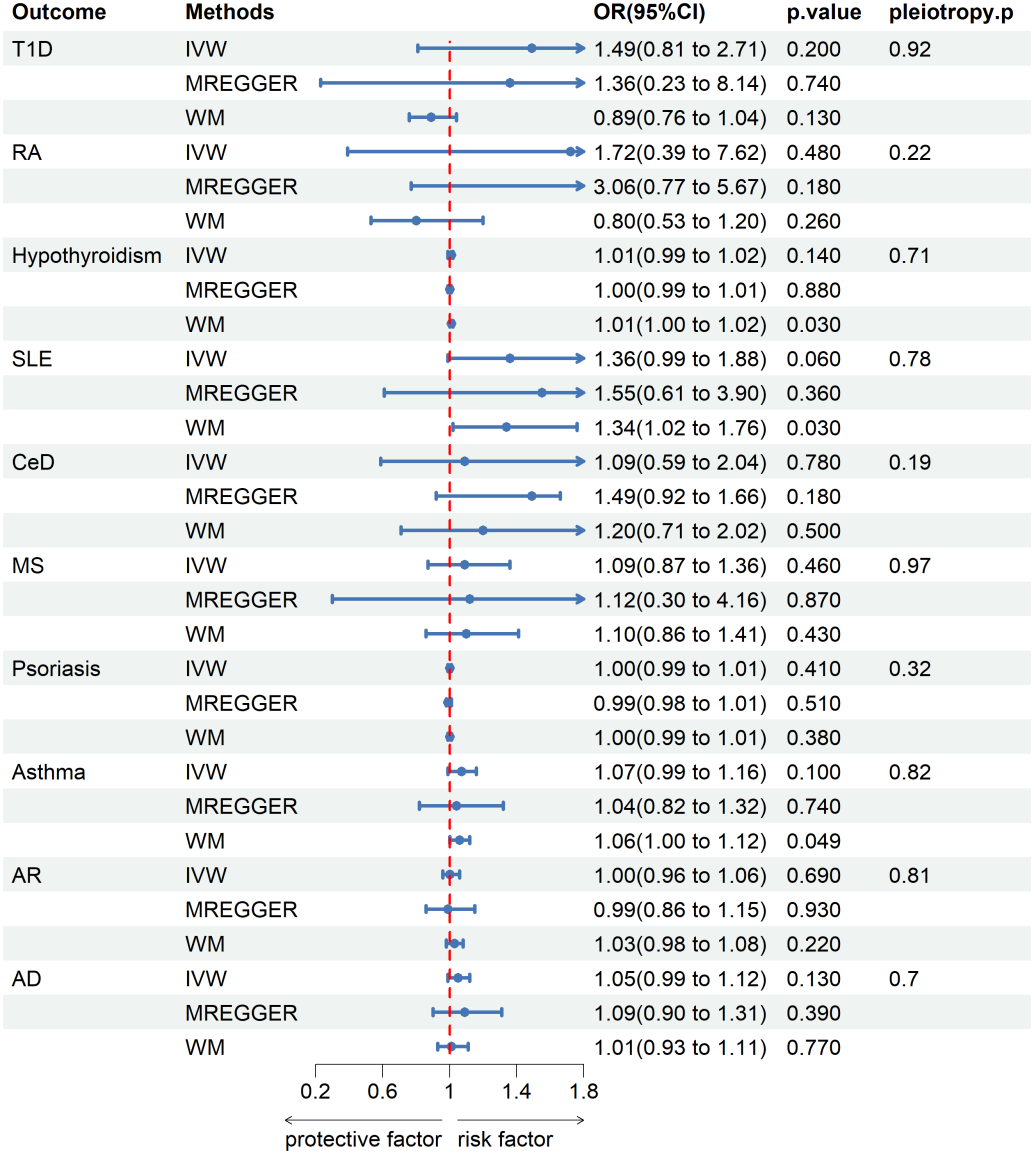
Mendelian randomization estimates from instrument variants for senile cataract on risk of autoimmune and allergic diseases.

No genetic predisposition to any of the 10 diseases was associated with senile cataract in the inverse analysis with IVs to senile cataract as exposure and the 10 diseases as outcomes (**Figure 3**).

### Sensitivity analysis

**Figure 4:**
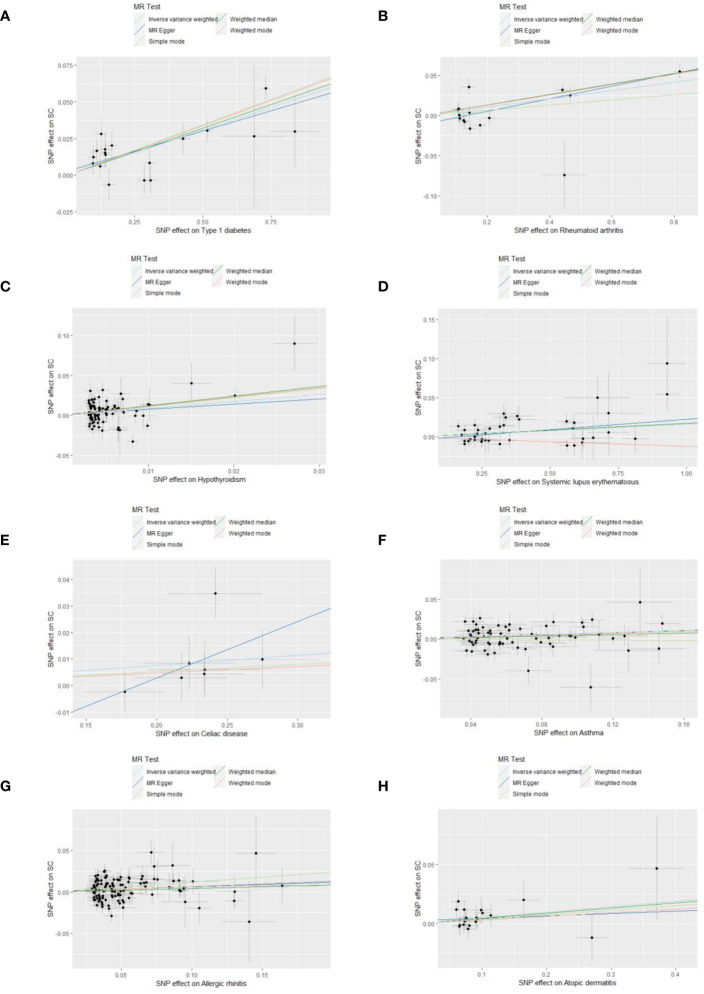
Scatter plots show the MR effect of each exposure on senile cataract in different MR methods. **(A)** Type 1 diabetes on senile cataract. **(B)** Rheumatoid arthritis on senile cataract. **(C)** Hypothyroidism on senile cataract. **(D)** Systemic lupus erythematosus on senile cataract. **(E)** Celiac disease on senile cataract. **(F)** Asthma on senile cataract. **(G)** Allergic rhinitis on senile cataract. **(H)** Allergic dermatitis on senile cataract.

**Figure 5:**
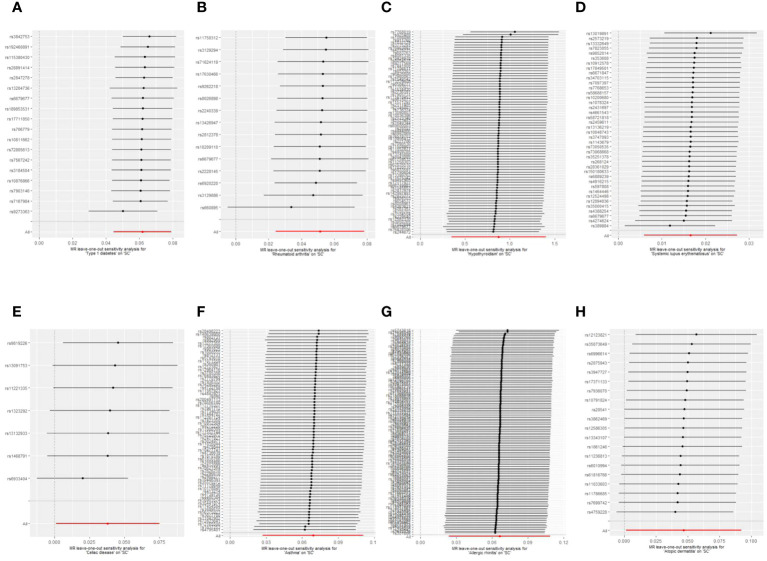
Leave-one-out plots of the causal relationships between autoimmune, allergic diseases and senile cataract. **(A)** Type 1 diabetes on senile cataract. **(B)** Rheumatoid arthritis on senile cataract. **(C)** Hypothyroidism on senile cataract. **(D)** Systemic lupus erythematosus on senile cataract. **(E)** Celiac disease on senile cataract. **(F)** Asthma on senile cataract. **(G)** Allergic rhinitis on senile cataract. **(H)** Allergic dermatitis on senile cataract.

Sensitivity analyses were performed to check the reliability of the IVW results. The effect of pleiotropy of exposures may be negligible given the intercept value, as no evidence of directional pleiotropy was found in the MR-Egger regression analysis (*p*>0.05) (**Table 2**). Cochrane's Q test showed that there was heterogeneity in MR analysis results between RA, hypothyroidism, SLE, psoriasis, asthma, AR with senile cataract (*p<*0.05) (**Table 2**). When the number of IVs is large, the existence of heterogeneity is unavoidable. Since we used random effects IVW as the primary analysis method, heterogeneity is acceptable and does not affect the estimation of causality (43). The MR-PRESSO test suggested that there were horizontal pleiotropic outliers for RA, hypothyroidism, MS, psoriasis and AR. The outliers-corrected results were shown in **Figure 2**. The results show that removing outliers did not affect the causal relationships implied by the main IVW results. Leave-one-out analysis results show that all SNPs are evenly distributed on the side of 0. It seems that no SNPs can strongly promote the overall effect of each exposure on senile cataract. The visualized scatter plots and Leave-one-out plots are shown in **Figures 4**, **5**.

**Table 2:** The results of sensitivity analysis.

Exposure	MRPRESSO *p*	Heterogeneity *p*	MR-Egger intercept	Pleiotropy *p*
Type 1 diabetes	0.107	0.09	0.00264	0.58
Rheumatoid arthritis	0.004*	1.18×10^-4^*	-0.0104	0.09
Hypothyroidism	<0.001*	5.27×10^-6^*	0.00135	0.66
Systemic lupus erythematosus	0.053	0.04*	-0.00398	0.35
Celiac disease	0.219	0.174	-0.0395	0.29
Multiple sclerosis	0.048*	0.06	0.00186	0.86
Psoriasis	0.021*	4.17×10^-4^*	-0.000765	0.14
Asthma	0.71	1.17×10^-3^*	0.000269	0.94
Allergic rhinitis	<0.001*	2.76×10^-5^*	0.000518	0.85
Atopic dermatitis	0.915	0.906	0.00609	0.69

MRPRESSO, MR pleiotropy residual sum and outlier; IVW, inverse variance weighted.

*p<0.05 was considered statistically significant.

## Discussion

In our study, a comprehensive bidirectional two-sample MR study was performed to investigate the causal associations between liabilities to ten diseases and the risk of senile cataract. MR analysis suggested a significant causal relationship between T1D, RA, hypothyroidism, SLE, asthma, AR and senile cataract. To the best of our knowledge, this is the first study to investigate the genetic causal links between autoimmune, allergic disease and senile cataract, making a significant contribution to understanding of the mechanism underlying senile cataract.

As a relatively common autoimmune disease, T1D is charactered by a complete lack of insulin due to the destruction of pancreatic beta cells, and insulin therapy must be given (44). Wen-Li Lu et al. reported that T1D patients had a higher risk of cataract compared to age- and sex-matched general population using cohort methodology in Taiwan (n=3,622) (13). Potential pathophysiological mechanisms may involve the aldose reductase pathway, oxidative stress, osmotic damage and autoimmunity (45). Papadimitriou et al. proposed an autoimmune hypothesis for acute bilateral cataract in T1D (24). Cataract formation typically occurs within weeks or months of the initiation of insulin therapy, coinciding with the time when insulin autoantibody became positive and the immunoreactivity of insulin receptors in the lens decreases. Further studies are necessary for clarification of these points and possibly for histological evidence of autoimmune processes (24). RA is a common autoimmune disease associated with hyperplasia of the joint tissues and the inflammation of the synovium, which can eventually lead to several serious systemic diseases, including pulmonary, cardiovascular, skeletal and psychological diseases (46). Eye diseases such as dry eye, glaucoma are common complications of RA, with a prevalence rate of about 18% (47). No related reports about RA increasing susceptibility to senile cataract directly were retrieved in PubMed database. However, in the treatment of RA, glucocorticoids (GCs) are commonly used. Posterior subcapsular cataract are known as side effects of long-term use of GCs (48), but it is still inconclusive whether the risk of cataract will increase during the treatment of RA with GCs (49). Our MR analysis provided genetic evidence that the onset of RA may increase the genetic susceptibility to cataract (OR=1.05). Primary hypothyroidism is defined by a high thyrotropin concentration along with low thyroid hormone concentrations or concentrations within the reference range. Hypothyroidism is usually an autoimmune disease in adults, which primarily affects middle-aged and elderly females (50). Limin Wei et al. published a case report that an East Asian 19-year-old male with Klinefelter Syndrome presenting cleft palate, hypothyroidism, cataract, and diabetes (51). This patient did not have congenital cataract because they were not diagnosed in infancy. Therefore, it is inferred that the formation of cataract in this patient may be related to hypothyroidism or diabetes, and there may be a potential genetic relationship between these diseases (51). Our results of MR analysis further verified this conjecture. SLE is a long-lasting autoimmune disease affecting multiple organs. It occurs when there is a failure in the regulation and tolerance of the immune system, affecting both the innate and adaptive immune responses (52). Cataract is the most prevalent ocular impairment in SLE. According to Alderaan et al., cataract development among patients with SLE is multifactorial and associated with the cumulative prednisone dose equivalent, systolic blood pressure and disease activity (12). Celiac disease, also known as gluten intolerance, is a condition that affects the small intestine and is characterized by an immune-mediated enteropathy. Ocular disease associated with celiac disease can sometimes be the first sign of the condition (53). Mollazadegan et al. conducted an European population-based cohort study (n=28756) and identified a moderate increase in the risk of cataract development among individuals diagnosed with biopsy-verified CeD (11). The cause of the condition has not been fully established, but research suggests that it may be related to a lack of absorption of vitamins and trace elements, dehydration, autoimmune factors and oxidative stress (11). Above findings supported the results obtained from our MR analyses, indicating that there is a positive association between genetically proxied autoimmune diseases and the risk of senile cataract.

Oxidative stress is the result of the imbalance between oxidant production and antioxidant defense mechanism. Reaction intermediates (free radicals and peroxides) that cannot be eliminated in time can have toxic effects on cellular components such as DNA, proteins, and lipids (54). The inflammatory response is associated with increased production of reactive oxygen species (ROS) and reactive nitrogen species (RNS), which is a shared characteristic among different autoimmune diseases, including RA, hypothyroidism, T1D, SLE, and others (55, 56). Lipid peroxidation caused by increased free radicals due to increased oxidative pressure of the lens or decreased ability to remove ROS is also an important pathological mechanism for cataract formation. Oxidative stress and autoimmunity-induced DNA damage, telomerase inhibition, and significant telomere shortening also accelerate aging (57, 58). Therefore, it can be inferred that the oxidative stress state of patients with autoimmune diseases may increase their susceptibility to senile cataract. A large amount of evidence suggests that autophagy deficiency is related to the development of autoimmunity. Meanwhile, the destruction of autophagy of lens cells can also lead to the loss of anti-stress ability and inhibit differentiation, and eventually lead to the formation of cataract (59, 60). Ferroptosis is a newly discovered type of iron-dependent programmed cell death characterized by excessive iron accumulation, elevated lipid peroxides, reactive oxygen species, reduced glutathione and glutathione peroxidase levels. A large and emerging literature on ferroptosis demonstrates the critical role of these pathological processes in autoimmune and ocular diseases (61). In addition, improper degradation of DNA during programmed death may also lead to autoimmune diseases and cataract (62).

Asthma is a chronic inflammatory disorder involving a large number of cells and cellular elements in the respiratory system (63). Li et al. found that cataracts had a positive correlation with asthma after adjusting for confounding factors by analyzing nationally representative samples from the National Health Interview Survey (NHIS) (16). Asthma may be closely related to cataracts through the PI3K-AKT-mTOR signaling pathway. In addition, allergy-induced inflammation or immune dysregulation is also one of the potential mechanisms leading to cataract formation (26). AR is a chronic inflammatory disease of the upper respiratory tract characterized by sneezing, itching, nasal congestion, and rhinorrhea (64). A Korean population-based survey reported that people with asthma and AR were more likely to develop senile cataracts but not with AD (17). Intranasal corticosteroids, a well-established and effective treatment for AR, does not appear to increase the risk of cataract in patients (65). The association between allergic diseases and cataracts may be attributed to multiple mechanisms, such as having similar risk factors, hypertension, hyperglycemia, etc. Other factors including inflammation and oxidative stress may also increase susceptibility to cataract (66). Our results showed that there is a suggestive genetic association between AD and senile cataract (p=0.0426). Therefore, as for whether AD increases the risk of senile cataract, based on our MR analysis and previous research results, we cannot determine the causal relationship between them, and further RCT is needed (17, 67).

A cause-and-effect relationship between the risk of senile cataract and MS, psoriasis could not be established in our study. Classified as an organ-specific, T-cell-mediated autoimmune disease, MS is the most common disabling disease in young adults that is not caused by physical trauma (68). A European population-based cohort study (n = 39,444) showing MS patients under the age of 50, especially young men, are at higher risk of developing cataract, compared with healthy controls (14). This result is contrary to the results of our MR analysis that there is no causal relationship between MS and the onset of cataract. Compared to observational studies, our MR study results are more compelling due to its ability to reduce confounding factors and reverse-causal association bias to some extent. Nonetheless, more advanced RCTs should be designed to confirm the causal association between the two diseases. Psoriasis, a polygenic disease characterized by erythematous plaques with silvery scales, is a chronic inflammatory autoimmune skin disorder affecting 1-3% of the world's population (69). Fuying Chen and colleagues have identified a new condition called the CAOP syndrome, which involves cataracts, alopecia, oral mucosal disorders and psoriasis-like symptoms (70). Contrary to our results, a population-based cohort by Chun-Yu Cheng has been observed a positive correlation between psoriasis and cataract. The common pathogenesis of cataract and psoriasis may be related to interleukin-6, C-reactive protein, intracellular adhesion molecule- 1, oxidative stress and so on (15). These differences in causality may be due to potential confounding factors, such as steroid hormone use and ultraviolet radiation, because observational studies are difficult to escape the influence of confounding factors. Therefore, there is likely no causal relationship between psoriasis and senile cataract based on MR analysis.

Senile cataract, as a reversible blinding eye disease with a high incidence rate, has caused great damage to labor productivity and brought a heavy economic burden around the world, especially in developing countries with in adequate surgical facilities. Understanding which factors increase the risk of cataracts can help identify high-risk individuals. Through the findings of this paper, people with autoimmune diseases should have regular anterior segment examinations through slit lamp microscopy while actively treating the primary disease, which will help ophthalmologists formulate preventive strategies in real time or select the most appropriate time for surgical treatment.

The main strength of our study is that we conducted an MR analysis and explored the genetic causal relationship between autoimmune, allergic diseases and the risk of senile cataract for the first time. Furthermore, various techniques were employed to perform sensitivity analysis, identifying outliers and correcting any potential pleiotropy and heterogeneity. With the increasing availability of a large amount of genetic data, the extension of GWAS may achieve early prediction of senile cataract and make it possible to achieve genetic-based treatment. The current study also has several limitations. Firstly, we used conventional methods that outcomes data were obtained from the FinnGen database and exposures data were obtained from other GWASs study on European ancestry in IEU Open GWAS database to reduce sample overlap. However, it is difficult to determine whether overlapping subjects were included in our MR analysis. Secondly, there is a possibility that a complete identification of all SNPs linked to these diseases was not achieved. By utilizing a restricted number of SNPs to establish the causal associations, it is possible that the statistical power of certain analyses may have been diminished. Thirdly, there may be potential confounding factors, such as the use of steroid hormone mediating the causality of autoimmune diseases on cataract, but due to the limitations of GWAS, we are not yet able to perform multivariate MR and mediation analysis. Last but not least, the data related to autoimmune, allergic diseases and senile cataracts were mostly from European ancestry. The results of our study should not be directly generalized to other ethnic groups.

## Conclusion

In summary, our current findings provide strong genetic evidence in support of the causal relationship among type 1 diabetes, rheumatoid arthritis, hypothyroidism, systemic lupus erythematosus, asthma, allergic rhinitis and senile cataract in a European population. Our study confirms previous observational studies, suggesting that autoimmune and allergy processes may be risk factors of senile cataract. The results of this study suggest that patients with autoimmune or allergy diseases should pay attention to the prevention and treatment of senile cataract and further studies are needed to clarify the potential role of autoimmune and allergy in the pathophysiology of senile cataract.

## Data availability statement

The original contributions presented in the study are included in the article/**Supplementary Material**. Further inquiries can be directed to the corresponding authors.

## Ethics statement

All analyses were based on publicly shared databases and no additional ethical approvals were required.

## Author contributions

WY: Writing – original draft, Software, Formal analysis, Conceptualization. XL: Writing – original draft, Visualization. GW: Writing – original draft, Resources. BQ: Writing – review & editing, Supervision. FZ: Writing – review & editing, Funding acquisition, Investigation, Supervision.

## References

[1] Hashemi H Pakzad R Yekta A Aghamirsalim M Pakbin M Ramin S. Global and regional prevalence of age-related cataract: a comprehensive systematic review and meta-analysis. Eye (Lond). (2020) 34:1357–70. doi: 10.1038/s41433-020-0806-3 PMC7376226 32055021

[2] He M Wang W and Huang W. Variations and trends in health burden of visual impairment due to cataract: A global analysis. Invest Ophthalmol Vis Sci. (2017) 58:4299–306. doi: 10.1167/iovs.17-21459 28846778

[3] Mitchell P Cumming RG Attebo K Panchapakesan J. Prevalence of cataract in Australia: the Blue Mountains eye study. Ophthalmology. (1997) 104:581–8. doi: 10.1016/S0161-6420(97)30266-8 9111249

[4] Yu X Lyu D Dong X He J and Yao K. Hypertension and risk of cataract: a meta-analysis. PloS One. (2014) 9:e114012. doi: 10.1371/journal.pone.0114012 25474403 PMC4256215

[5] Ye J He J Wang C Wu H Shi X Zhang H. Smoking and risk of age-related cataract: a meta-analysis. Invest Ophthalmol Vis Sci. (2012) 53:3885–95. doi: 10.1167/iovs.12-9820 22599585

[6] Chua SYL Luben RN Hayat S Broadway DC Khaw KT Warwick A. Alcohol consumption and incident cataract surgery in two large UK cohorts. Ophthalmology. (2021) 128:837–47. doi: 10.1016/j.ophtha.2021.02.007 PMC8162662 33571551

[7] Pek A Szabo D Sandor GL Toth G Papp A Nagy ZZ. Relationship between diabetes mellitus and cataract in Hungary. Int J Ophthalmol. (2020) 13:788–93. doi: 10.18240/ijo PMC7201352 32420227

[8] Jacobson DL Gange SJ Rose NR and Graham NM. Epidemiology and estimated population burden of selected autoimmune diseases in the United States. Clin Immunol Immunopathol. (1997) 84:223–43. doi: 10.1006/clin.1997.4412 9281381

[9] Wang L Wang FS and Gershwin ME. Human autoimmune diseases: a comprehensive update. J Intern Med. (2015) 278:369–95. doi: 10.1111/joim.12395 26212387

[10] Nwaru BI Virtanen SM. Allergenic food introduction and childhood risk of allergic or autoimmune disease. JAMA. (2017) 317:86. doi: 10.1001/jama.2016.18329 28030695

[11] Mollazadegan K Kugelberg M Lindblad BE Ludvigsson JF. Increased risk of cataract among 28,000 patients with celiac disease. Am J Epidemiol. (2011) 174:195–202. doi: 10.1093/aje/kwr069 21624959

[12] Alderaan K Sekicki V Magder LS and Petri M. Risk factors for cataracts in systemic lupus erythematosus (SLE). Rheumatol Int. (2015) 35:701–8. doi: 10.1007/s00296-014-3129-5 25257763

[13] Lu WL Shen PC Lee CH Su YT and Chen LM. High risk of early cataracts in young type 1 diabetes group: A nationwide cohort study. Int J Endocrinol. (2020) 2020:8160256. doi: 10.1155/2020/8160256 33133186 PMC7568800

[14] Bazelier MT Mueller-Schotte S Leufkens HG Uitdehaag BM van Staa T de Vries F. Risk of cataract and glaucoma in patients with multiple sclerosis. Mult Scler. (2012) 18:628–38. doi: 10.1177/1352458511426737 22025330

[15] Cheng CY. Risk of incident cataract in patients with psoriasis: A population-based cohort study. J Dermatol. (2022) 49:359–67. doi: 10.1111/1346-8138.16261 34862667

[16] Li W Wang B. Cross-sectional study of the association between asthma and cataract among 40 years and older in the USA. BMC Ophthalmol. (2022) 22:340. doi: 10.1186/s12886-022-02564-y 35948897 PMC9364527

[17] Lee YB Lee JH Kang MJ Choi JY Kim JW Yu DS. Association between allergic diseases and ophthalmologic diseases, including cataracts and glaucoma, using the Korean National Health and Nutrition Examination Survey 2010-2012: A STROBE-compliant article. J Dermatol. (2018) 45:463–7. doi: 10.1111/1346-8138.14193 29315741

[18] Skrivankova VW Richmond RC Woolf BAR Yarmolinsky J Davies NM Swanson SA. Strengthening the reporting of observational studies in epidemiology using mendelian randomization: the STROBE-MR statement. JAMA. (2021) 326:1614–21. doi: 10.1001/jama.2021.18236 34698778

[19] Smith GD Ebrahim S. 'Mendelian randomization': can genetic epidemiology contribute to understanding environmental determinants of disease? Int J Epidemiol. (2003) 32:1–22. doi: 10.1093/ije/dyg070 12689998

[20] Zheng J Baird D Borges MC Bowden J Hemani G Haycock P. Recent developments in mendelian randomization studies. Curr Epidemiol Rep. (2017) 4:330–45. doi: 10.1007/s40471-017-0128-6 PMC5711966 29226067

[21] Mokry LE Ahmad O Forgetta V Thanassoulis G Richards JB. Mendelian randomisation applied to drug development in cardiovascular disease: a review. J Med Genet. (2015) 52:71–9. doi: 10.1136/jmedgenet-2014-102438 25515070

[22] Liu YC Wilkins M Kim T Malyugin B and Mehta JS. Cataracts. Lancet. (2017) 390:600–12. doi: 10.1016/S0140-6736(17)30544-5 28242111

[23] Vinson JA. Oxidative stress in cataracts. Pathophysiology. (2006) 13:151–62. doi: 10.1016/j.pathophys.2006.05.006 16765571

[24] Papadimitriou DT Bothou C Skarmoutsos F Alexandrides TK Papaevangelou V Papadimitriou A. The autoimmune hypothesis for acute bilateral cataract in type 1 diabetes. Diabetes Metab. (2016) 42:386–7. doi: 10.1016/j.diabet.2016.04.006 27209440

[25] Ranjan M Nayak S Kosuri T and Rao BS. Immunochemical detection of glycated lens crystallins and their circulating autoantibodies in human serum during aging. Mol Vis. (2008) 14:2056–66. PMC2584771 19023447

[26] Zhao Y Li X Xu Z Hao L Zhang Y and Liu Z. PI3K-AKT-mTOR signaling pathway: the intersection of allergic asthma and cataract. Pharmazie. (2019) 74:598–600. doi: 10.1691/ph.2019.9080 31685084

[27] Sakaue S Kanai M Tanigawa Y Karjalainen J Kurki M Koshiba S. A cross-population atlas of genetic associations for 220 human phenotypes. Nat Genet. (2021) 53:1415–24. doi: 10.1038/s41588-021-00931-x PMC12208603 34594039

[28] Eyre S Bowes J Diogo D Lee A Barton A Martin P. High-density genetic mapping identifies new susceptibility loci for rheumatoid arthritis. Nat Genet. (2012) 44:1336–40. doi: 10.1038/ng.2462 PMC3605761 23143596

[29] Dubois PC Trynka G Franke L Hunt KA Romanos J Curtotti A. Multiple common variants for celiac disease influencing immune gene expression. Nat Genet. (2010) 42:295–302. doi: 10.1038/ng.543 20190752 PMC2847618

[30] Bentham J Morris DL Graham DSC Pinder CL Tombleson P Behrens TW. Genetic association analyses implicate aberrant regulation of innate and adaptive immunity genes in the pathogenesis of systemic lupus erythematosus. Nat Genet. (2015) 47:1457–64. doi: 10.1038/ng.3434 PMC4668589 26502338

[31] The International Multiple Sclerosis Genetics Consortium & The Wellcome Trust Case Control Consortium 2. Genetic risk and a primary role for cell-mediated immune mechanisms in multiple sclerosis. Nature. (2011) 476:214–9. doi: 10.1038/nature10251 PMC3182531 21833088

[32] Valette K Li Z Bon-Baret V Chignon A Berube JC Eslami A. Prioritization of candidate causal genes for asthma in susceptibility loci derived from UK Biobank. Commun Biol. (2021) 4:700. doi: 10.1038/s42003-021-02227-6 34103634 PMC8187656

[33] Mbatchou J Barnard L Backman J Marcketta A Kosmicki JA Ziyatdinov A. Computationally efficient whole-genome regression for quantitative and binary traits. Nat Genet. (2021) 53:1097–103. doi: 10.1038/s41588-021-00870-7 34017140

[34] Sliz E Huilaja L Pasanen A Laisk T Reimann E Magi R. Uniting biobank resources reveals novel genetic pathways modulating susceptibility for atopic dermatitis. J Allergy Clin Immunol. (2022) 149:1105–12 e9. doi: 10.1016/j.jaci.2021.07.043 34454985

[35] Bowden J Davey Smith G Haycock PC and Burgess S. Consistent estimation in mendelian randomization with some invalid instruments using a weighted median estimator. Genet Epidemiol. (2016) 40:304–14. doi: 10.1002/gepi.21965 PMC4849733 27061298

[36] Kurki MI Karjalainen J Palta P Sipila TP Kristiansson K Donner KM. FinnGen provides genetic insights from a well-phenotyped isolated population. Nature. (2023) 613:508–18. doi: 10.1038/s41586-022-05473-8 PMC9849126 36653562

[37] Burgess S Butterworth A Thompson SG. Mendelian randomization analysis with multiple genetic variants using summarized data. Genet Epidemiol. (2013) 37:658–65. doi: 10.1002/gepi.21758 PMC4377079 24114802

[38] Bowden J Davey Smith G Burgess S. Mendelian randomization with invalid instruments: effect estimation and bias detection through Egger regression. Int J Epidemiol. (2015) 44:512–25. doi: 10.1093/ije/dyv080 PMC4469799 26050253

[39] Zhu Z Zhang F Hu H Bakshi A Robinson MR Powell JE. Integration of summary data from GWAS and eQTL studies predicts complex trait gene targets. Nat Genet. (2016) 48:481–7. doi: 10.1038/ng.3538 27019110

[40] Verbanck M Chen CY Neale B and Do R. Detection of widespread horizontal pleiotropy in causal relationships inferred from Mendelian randomization between complex traits and diseases. Nat Genet. (2018) 50:693–8. doi: 10.1038/s41588-018-0099-7 PMC6083837 29686387

[41] Bowden J Del Greco MF Minelli C Davey Smith G Sheehan N and Thompson J. A framework for the investigation of pleiotropy in two-sample summary data Mendelian randomization. Stat Med. (2017) 36:1783–802. doi: 10.1002/sim.7221 PMC5434863 28114746

[42] Emdin CA Khera AV and Kathiresan S. Mendelian randomization. JAMA. (2017) 318:1925–6. doi: 10.1001/jama.2017.17219 29164242

[43] Burgess S Davey Smith G Davies NM Dudbridge F Gill D Glymour MM. Guidelines for performing Mendelian randomization investigations: update for summer 2023. Wellcome Open Res. (2019) 4:186. doi: 10.12688/wellcomeopenres 32760811 PMC7384151

[44] Katsarou A Gudbjornsdottir S Rawshani A Dabelea D Bonifacio E Anderson BJ. Type 1 diabetes mellitus. Nat Rev Dis Primers. (2017) 3:17016. doi: 10.1038/nrdp.2017.16 28358037

[45] Obrosova IG Chung SS and Kador PF. Diabetic cataracts: mechanisms and management. Diabetes Metab Res Rev. (2010) 26:172–80. doi: 10.1002/dmrr.1075 20474067

[46] McInnes IB Schett G. The pathogenesis of rheumatoid arthritis. N Engl J Med. (2011) 365:2205–19. doi: 10.1056/NEJMra1004965 22150039

[47] Turk MA Hayworth JL Nevskaya T and Pope JE. Ocular manifestations in rheumatoid arthritis, connective tissue disease, and vasculitis: A systematic review and metaanalysis. J Rheumatol. (2021) 48:25–34. doi: 10.3899/jrheum.190768 32358156

[48] Black RL Oglesby RB Von Sallmann L and Bunim JJ. Posterior subcapsular cataracts induced by corticosteroids in patients with rheumatoid arthritis. JAMA. (1960) 174:166–71. doi: 10.1001/jama.1960.63030020005014 13801171

[49] Black RJ Hill CL Lester S and Dixon WG. The association between systemic glucocorticoid use and the risk of cataract and glaucoma in patients with rheumatoid arthritis: A systematic review and meta-analysis. PloS One. (2016) 11:e0166468. doi: 10.1371/journal.pone.0166468 27846316 PMC5112962

[50] Iglesias P Bajo MA Selgas R and Diez JJ. Thyroid dysfunction and kidney disease: An update. Rev Endocr Metab Disord. (2017) 18:131–44. doi: 10.1007/s11154-016-9395-7 27864708

[51] Wei L Liu Y Sun S Tang Y Chen S and Song G. Case report of 49,XXXXY syndrome with cleft palate, diabetes, hypothyroidism, and cataracts. Med (Baltimore). (2019) 98:e17342. doi: 10.1097/MD.0000000000017342 PMC6775424 31574874

[52] Tsokos GC Lo MS Costa Reis P Sullivan KE. New insights into the immunopathogenesis of systemic lupus erythematosus. Nat Rev Rheumatol. (2016) 12:716–30. doi: 10.1038/nrrheum.2016.186 27872476

[53] Fousekis FS Katsanos A Katsanos KH Christodoulou DK. Ocular manifestations in celiac disease: an overview. Int Ophthalmol. (2020) 40:1049–54. doi: 10.1007/s10792-019-01254-x 31916055

[54] Ramani S Pathak A Dalal V Paul A and Biswas S. Oxidative stress in autoimmune diseases: an under dealt Malice. Curr Protein Pept Sci. (2020) 21:611–21. doi: 10.2174/1389203721666200214111816 32056521

[55] Ruggeri RM Campenn IA Giuffrida G Casciaro M Barbalace MC Hrelia S. Oxidative stress as a key feature of autoimmune thyroiditis: an update. Minerva Endocrinol. (2020) 45:326–44. doi: 10.23736/S0391-1977.20.03268-X 32969631

[56] Smallwood MJ Nissim A Knight AR Whiteman M Haigh R and Winyard PG. Oxidative stress in autoimmune rheumatic diseases. Free Radic Biol Med. (2018) 125:3–14. doi: 10.1016/j.freeradbiomed.2018.05.086 29859343

[57] Babizhayev MA Yegorov YE. Telomere attrition in lens epithelial cells - a target for N-acetylcarnosine therapy. Front Biosci (Landmark Ed). (2010) 15:934–56. doi: 10.2741/3655 20515735

[58] Weyand CM Goronzy JJ. The immunology of rheumatoid arthritis. Nat Immunol. (2021) 22:10–8. doi: 10.1038/s41590-020-00816-x PMC8557973 33257900

[59] Keller CW Adamopoulos IE Lunemann JD. Autophagy pathways in autoimmune diseases. J Autoimmun. (2023) 136:103030. doi: 10.1016/j.jaut.2023.103030 37001435 PMC10709713

[60] Chai P Ni H Zhang H and Fan X. The evolving functions of autophagy in ocular health: A double-edged sword. Int J Biol Sci. (2016) 12:1332–40. doi: 10.7150/ijbs.16245 PMC5118779 27877085

[61] Liu K Li H Wang F and Su Y. Ferroptosis: mechanisms and advances in ocular diseases. Mol Cell Biochem. (2023) 478:2081–95. doi: 10.1007/s11010-022-04644-5 36617346

[62] Nagata S. DNA degradation in development and programmed cell death. Annu Rev Immunol. (2005) 23:853–75. doi: 10.1146/annurev.immunol.23.021704.115811 15771588

[63] Asher MI Montefort S Bjorksten B Lai CK Strachan DP Weiland SK. Worldwide time trends in the prevalence of symptoms of asthma, allergic rhinoconjunctivitis, and eczema in childhood: ISAAC Phases One and Three repeat multicountry cross-sectional surveys. Lancet. (2006) 368:733–43. doi: 10.1016/S0140-6736(06)69283-0 16935684

[64] Skoner DP. Allergic rhinitis: definition, epidemiology, pathophysiology, detection, and diagnosis. J Allergy Clin Immunol. (2001) 108:S2–8. doi: 10.1067/mai.2001.115569 11449200

[65] Valenzuela CV Liu JC Vila PM Simon L Doering M and Lieu JEC. Intranasal corticosteroids do not lead to ocular changes: A systematic review and meta-analysis. Laryngoscope. (2019) 129:6–12. doi: 10.1002/lary.27209 30229924 PMC6320292

[66] Paik JS Han K Nam G Park SK Hwang HS Chun YH. Increased risk of cataract surgery in patients with allergic disease: a population based cohort study. Sci Rep. (2022) 12:21258. doi: 10.1038/s41598-022-25589-1 36482171 PMC9732285

[67] Rim TH Kim DW Kim SE and Kim SS. Factors associated with cataract in korea: A community health survey 2008-2012. Yonsei Med J. (2015) 56:1663–70. doi: 10.3349/ymj.2015.56.6.1663 PMC4630058 26446652

[68] Dobson R Giovannoni G. Multiple sclerosis - a review. Eur J Neurol. (2019) 26:27–40. doi: 10.1111/ene.13819 30300457

[69] Chandran NS Greaves M Gao F Lim L and Cheng BC. Psoriasis and the eye: prevalence of eye disease in Singaporean Asian patients with psoriasis. J Dermatol. (2007) 34:805–10. doi: 10.1111/j.1346-8138.2007.00390.x 18078405

[70] Chen F Ni C Wang X Cheng R Pan C Wang Y. S1P defects cause a new entity of cataract, alopecia, oral mucosal disorder, and psoriasis-like syndrome. EMBO Mol Med. (2022) 14:e14904. doi: 10.15252/emmm.202114904 35362222 PMC9081911

