# Supplementary material for: Association of autoimmune and allergic diseases with senile cataract: a bidirectional two-sample Mendelian randomization study
Source: Front Immunol. 2024 Mar 22;15:1325868. doi: 10.3389/fimmu.2024.1325868 (PMC10995295; doi:10.3389/fimmu.2024.1325868)
Supplement: Supplementary file 1 [file Table_1.docx]

**Supplementary Material**

**Title** Association of autoimmune and allergic diseases with senile cataract A Bidirectional Two-Sample Mendelian Randomization Study

**Authors** Weichen Yuan, Xiangrui Li, Guan Wang, Fangkun Zhao

**Supplementary Tables**

**Supplementary Table S1. STROBE-MR checklist**

| **Item** | **Complete/location** |
| --- | --- |
| **1.** **Title** **and** **Abstract:** "Mendelian randomization" is named both in the title and the abstract | Complete |
| **Introduction** |  |
| **2.** **Background:** Explain the scientific background and rationale for the reported study. Is causality between exposure and outcome plausible? Justify why MR is a helpful method to address the study question. | Concept of Mendelian randomization and specific request for Mendelian randomization were explained in the second and third paragraph of the introduction (**Page 4**). |
| **3.** **Objectives:** State specific objectives clearly, including pre-specified causal hypotheses (if any). | The causal question has been stated in the fourth paragraph of the introduction (**Page 5**). |
| **Methods** |  |
| **4.** **Study** **design** **and** **data** **sources:** Present key elements of study design early in the paper. Consider including a table listing sources of data for all phases of the study. For each data source contributing to the analysis, describe the following:  a) Describe the study design and the underlying population from which it was drawn. Describe also the setting, locations, and relevant dates, including periods of recruitment, exposure, follow-up, and data collection, if available.  b) Give the eligibility criteria, and the sources and methods of selection of participants.  c) Explain how the analyzed sample size was arrived at.  d) Describe measurement, quality and selection of genetic variants.  e) For each exposure, outcome and other relevant variables, describe methods of assessment and, in the case of diseases, the diagnostic criteria used.  f) Provide details of ethics committee approval and participant informed consent, if relevant. | All necessary information about the GWAS studies been used in this study have been described in the method section. The genetic predictor selection process has been described in in the Methods section “**Study Design**” (**Page 5**).  Ethics approval and informed consent info in the method section (**Page 7**). |
| **5.** **Assumptions:** Explicitly state assumptions for the main analysis (e.g. relevance, exclusion, independence, homogeneity) as well assumptions for any additional or sensitivity analysis. | All necessary information is described in the methods section “MR assumptions and Study Design” (**Page 5**). |
| **6.** **Statistical** **methods** **main** **analysis** Describe statistical methods and statistics used.  a) Describe how quantitative variables were handled in the analyses (i.e., scale, units, model).  b) Describe the process for identifying genetic variants and weights to be included in the analyses (i.e, independence and model). Consider a flow diagram.  c) Describe the MR estimator, e.g. two-stage least squares, Wald ratio, and related statistics. Detail the included covariates and, in case of two-sample MR, whether the same covariate set was used for adjustment in the two samples.  d) Explain how missing data were addressed.  e) If applicable, say how multiple testing was dealt with. | All necessary information is described in the methods section “Statistical analysis” (**Page 7** and **Page 8**). |
| **7.** **Assessment** **of** **assumptions:** **Describe any** **methods** **used** **to** **assess** **the** **assumptions** **or justify** **their** **validity.** | The relevance assumption was validated using the strength of the genetic predictors by F statistics (**Page 6**).  The exclusion restriction assumption was tested using the following sensitivity approaches: MR Egger regression and weighted median analysis (**Page 7**). |
| **8.** **Sensitivity** **analyses:** Describe any sensitivity analyses or additional analyses performed. | All necessary information is described in the methods section “Sensitivity analysis” **Page 8**). |
| **9.** **Software** **and** **pre-registration**  a) Name statistical software and package(s), including version and settings used.  b) State whether the study protocol and details were pre-registered (as well as when and where). | a) All statistical software and settings used are described in the method section (**Page 7**).  b) The analysis plan was described in the " Study design” section of the Methods (**Page 5**). |
| **Results** |  |
| **10.** **Descriptive** **data**  a) Report the numbers of individuals at each stage of included studies and reasons for exclusion. Consider use of a flow-diagram.  b) Report summary statistics for phenotypic exposure(s), outcome(s) and other relevant variables  (e.g. means, standard deviations, proportions).  c) If the data sources include meta-analyses of previous studies, provide the number of studies, their reported ancestry, if available, and assessments of heterogeneity across these studies. Consider using a supplementary table for each data source.  d) For two-sample Mendelian randomization:  i. Provide information on the similarity of the genetic variant-exposure associations between the exposure and outcome samples. ii. Provide information on extent of sample overlap between the exposure and outcome data sources. | a) Information is given in the " Study design" section of the Methods (**Page 6**).  b) We described the detailed information of the summary statistics for our analysis in **Table 1**.   c) NA.  d) We provide this information in the section of the Methods (**Page 7**). |
| **11.** **Main** **results**  a) Report the associations between genetic variant and exposure, and between genetic variant and outcome, preferably on an interpretable (e.g. comparing 25th and 75th percentile of allele counts or genetic risk score, if individual-level data available).  b) Report causal effect estimate between exposure and outcome, and the measures of uncertainty from the MR analysis. Use an intuitive scale, such as odds ratio, or relative risk, per standard deviation difference.  c) If relevant, consider translating estimates of relative risk into absolute risk for a meaningful time-period.  d) Consider any plots to visualize results (e.g. forest plot, scatter plot of associations between genetic variants and outcome versus between genetic variants and exposure). | a) Genetic exposure associations have been reported in **Data sheet**.  b) The causal effect estimates between exposures and outcomes and plots were listed in **Figure 2, 3, 4**, **5**. Our results were presented in terms of odds ratio and confidence intervals throughout the results section for binary outcomes and as beta coefficient for quantitative outcomes. |
| **12.** **Assessment** **of** **assumptions**  a) Assess the validity of the assumptions.  b) Report any additional statistics (e.g., assessments of heterogeneity, such as I2, Q statistic). | a) We assess the validity using sensitivity analyses, MR Egger regression and weighted median approach. Results were presented in **Fig. 2** and **Fig. 3**.  b) We presented the use of Egger intercept and Cochran's Q in the Results **Table 2**. |
| **13.** **Sensitivity** **and** **additional** **analyses**  a) Use sensitivity analyses to assess the robustness of the main results to violations of the assumptions.  b) Report results from other sensitivity analyses (e.g., replication study with different dataset, analyses of subgroups, validation of instrument(s),simulations, etc.).  c) Report any assessment of direction of causality (e.g., bidirectional MR).  d) When relevant, report and compare with estimates from non-MR analyses.  e) Consider any additional plots to visualize results(e.g., leave-one-out analyses). | a) We assess the robustness of the main results using Egger intercept, Cochran's Q, MR-PRESSO and Leave-one-out analyses. Results were presented in **Table 2, Fig. 4 and Fig. 5.**  c) Bidirectional MR results were shown in **Fig. 3**.  e) The results of leave-one-out analyses were shown in **Fig. 5**. |
| **Discussion** |  |
| **14.** **Key** **results** | Discussion paragraph 1 (**Page 10**) |
| **15.** **Limitations** Discuss limitations of the study, taking into account the validity of the MR assumptions, other sources of potential bias, and imprecision. Discuss both direction and magnitude of any potential bias, and any efforts to address them. | Study limitations (**Page 15**) |
| **16.** **Interpretations**  a) Give a cautious overall interpretation of results considering objectives and limitations. Compare with results from other relevant studies.  b) Discuss underlying biological mechanisms that could be modelled by using the genetic variants to assess the relationship between the exposure and the outcome.  c) Discuss whether the results have clinical or policy relevance, and whether interventions could have the same size effect. | a) Interpretation: Discussion paragraphs 2, 3, 4, 5 (**Page 10 -14**)  b) Underlying biological mechanisms: Discussion paragraphs 3 (**Page 12**).  c) Clinical practice: Discussion paragraphs 6 (**Page 14**) |
| **17.** **Generalizability:** | We have discussed the potential caveats in terms of generalizability of our findings in the Discussion section (**Page 15**). |
| **18.** **Funding:** | We have reported all sources of funding in the “Funding” section. |
| **19.** **Data** **and** **data** **sharing:** | We have provided the link/approach to access genetic data used in this study in the "Data Availability Statement" section. |
| **20.** **Conflicts** **of** **Interest:** | We have declared conflicts of interest in the "Disclosure" section. |
